# Multisensory Stimulation interventio Based on Snoezelen Principles for Elderly Peolple with Dementia a Pilot Study

**DOI:** 10.1002/alz70858_101116

**Published:** 2025-12-25

**Authors:** Gabriella Pereira Pilon, Marcia Maria Pires Camargo Novelli

**Affiliations:** ^1^ UNIFESP, Santos, São Paulo, Brazil; ^2^ Federal University of São Paulo, Santos, São Paulo, Brazil

## Abstract

**Background:**

Individuals with dementia may exhibit behavioral disturbances or behavioral and psychological symptoms (BPSD). Experimental and clinical trials suggest that Multisensory Stimulation (MSE), an intervention that applies sensory modulation resources and does not depend on verbal interactions, is a powerful tool for managing BPSD. However, there are no Brazilian studies that evaluate the benefits of MSE interventions in elderly people with dementia and BPSD living in the community. In this study, it was developed, implemented, and compared pre and post‐intervention in an outpatient intervention protocol for MSE in elderly people with dementia living in Brazilian society.

**Method:**

The MSE intervention was organized in 12 individual sessions, 45 minutes, once a week. There was a sample of 10 elderly people with dementia syndrome, splitted in an experimental group (*n* = 5) and a control group (*n* = 5). The intervention benefits were analyzed with objective measures using the Neuropsychiatric Inventory (NPI) and Subjective Perception of BPSD Improvement Scale. Therefore, subjective perspectives were collected about the intervention effects on behavioral and psychological symptoms in dementia (BPSD). Descriptive and comparative analysis were considered by the pre and post‐intervention and also the effect size.

**Result:**

Comparing the pre and post‐intervention using the NPI, there was an improvement in the EG and a worsening in the CG in all variables: Total score on the NPI (Cohen's d = ‐1.55), Frequency (Cohen's d = ‐1.12), Intensity (Cohen's *d* = ‐1.42), Caregiver exhaustion (Cohen's *d* = ‐ 1.19) and number of behaviors (Cohen's *d* = ‐0.55), adoption *p*‐value≤0.05.

**Conclusion:**

The MSE Intervention based on Snoezelen's principles has a positive effect on reducing the frequency and intensity of BPSD in elderly participants with dementia, as well as on the level of distress on caregivers related to these behaviors. These findings can help to better understand the effect of these non pharmacological interventions in older adults with dementia and BPSD living in the Brazilian community. This study is original and pioneering, providing valuable scientific evidence to the Brazilian community. Research on this topic remains limited, highlighting the need for further investigation to achieve more conclusive and in‐depth results.

